# Evaluation of corneal biomechanical properties using the ocular response analyzer and the dynamic Scheimpflug-Analyzer Corvis ST in high pressure and normal pressure open-angle glaucoma patients

**DOI:** 10.1371/journal.pone.0281017

**Published:** 2023-01-26

**Authors:** Lisa Ramm, Robert Herber, Georg Lorenz, Carolin S. Jasper, Lutz E. Pillunat, Karin R. Pillunat

**Affiliations:** 1 Department of Ophthalmology, University Hospital Carl Gustav Carus, TU Dresden, Dresden, Germany; 2 Faculty of Medicine Carl Gustav Carus, Department of Ophthalmology, TU Dresden, Dresden, Germany; University of North Texas Health Science Center, UNITED STATES

## Abstract

**Purpose:**

To characterize differences in corneal biomechanics in high (HPG) and normal pressure (NPG) primary open-angle glaucoma, and its association to disease severity.

**Methods:**

Corneal biomechanical properties were measured using the Ocular Response Analyzer (ORA) and the dynamic Scheimpflug-Analyzer Corvis ST (CST). Disease severity was functionally assessed by automated perimetry (Humphrey field analyzer) and structurally with the Heidelberg Retina Tomograph. To avoid a possible falsification by intraocular pressure, central corneal thickness and age, which strongly influence ORA and CST measurements, group matching was performed. Linear mixed models and generalized estimating equations were used to consider inter-eye correlation.

**Results:**

Following group matching, 60 eyes of 38 HPG and 103 eyes of 60 NPG patients were included. ORA measurement revealed a higher CRF in HPG than in NPG (P < 0.001). Additionally, the CST parameter integrated radius (P < 0.001) was significantly different between HPG and NPG. The parameter SSI (P < 0.001) representing corneal stiffness was higher in HPG than in NPG. Furthermore, regression analysis revealed associations between biomechanical parameters and indicators of disease severity. In HPG, SSI correlated to RNFL thickness. In NPG, dependencies between biomechanical readings and rim area, MD, and PSD were shown.

**Conclusion:**

Significant differences in corneal biomechanical properties were detectable between HPG and NPG patients which might indicate different pathophysiological mechanisms underlying in both entities. Moreover, biomechanical parameters correlated to functional and structural indices of diseases severity. A reduced corneal deformation measured by dynamic methods was associated to advanced glaucomatous damage.

## Introduction

Primary open-angle glaucoma (POAG) is characterized by a progressive loss of retinal ganglion cells and an irreversible axon degeneration in the optic nerve leading to visual field defects up to severe vision loss. Despite its great clinical significance, the pathogenesis of the disease has not been fully clarified [1, 2]. An increase in intraocular pressure (IOP) is the main risk factor for POAG development and progression [1, 3], but also further mechanisms seem to play an important role. Possible aspects in the pathogenesis of the glaucomatous optic neuropathy are genetic components, autoimmunity, failures in axonal transport and lack of trophic factors, changes in electrical activity of cells and glutamate excitotoxicity [2, 4]. Furthermore, disturbance in retinal and optic nerve head (ONH) blood supply may be important. Vascular dysregulation and impaired neurovascular coupling, reduced perfusion pressure, vasospasm, formation of reactive oxygen species as well as a mechanical vascular compression may be involved [5–8]. In regard to the assumption of a mechanical vascular compression, chronic remodeling of the ONH connective tissue with progredient glaucomatous cupping [9, 10] could be an important mechanism. Thereby, the association between a reduction in central corneal thickness (CCT) and glaucoma progression [3] as well as changes in corneal biomechanical properties in POAG patients [11–13] may indicate, that structural changes are not limited to the lamina cribrosa (LC), but rather affect the whole globe. In conformance, earlier studies reported stiffness changes of the sclera, the LC, the trabecular meshwork (TM) and the cornea [14–17]. Increased stiffness of the LC and the peripapillary sclera may result in a reduced compliance at the ONH with a higher susceptibility to IOP-induced glaucomatous injury [14, 18]. Since direct accessibility of the LC, the sclera and the ONH is limited measurements of corneal biomechanical properties may serve as an indicator for structural ONH integrity [19, 20]. The ocular response analyzer (ORA; Reichert Inc., Depew NY, USA) and the dynamic Scheimpflug-Analyzer Corvis ST (CST; Oculus Optikgeräte GmbH, Wetzlar, Germany) offer the opportunity of a direct, non-invasive assessment of corneal biomechanical properties.

The aim of the study was to characterize glaucomatous changes in corneal biomechanics as a potential indicator of tissue remodeling of the whole globe, and to investigate its association with disease severity in POAG patients. Special attention was paid to differences between high pressure (HPG) and normal pressure glaucoma (NPG), thereby contributing to the understanding of the pathogenesis of glaucomatous optic neuropathy.

## Methods

This prospective, cross-sectional study was conducted at the Department of Ophthalmology, University Hospital Carl Gustav Carus, TU Dresden, Germany between January 2016 and July 2019. The study protocol adhered to the tenets of the Declaration of Helsinki and was approved by the local ethics committee of the University of Dresden (registered at clinicaltrails.gov: NCT02959242). Patients with POAG admitted for a one-day in hospital routine glaucoma work up were consecutively included. Exclusion criteria were any preexisting corneal disease, ocular surgery in the past, diabetes mellitus, contact lens wear, systemic connective tissue diseases, pseudoexfoliation or other possible causes of secondary glaucoma and lacking capacity to consent.

The diagnosis of POAG was based on an open angle on gonioscopy, the presence of damage to the inner retinal layers on optical coherence tomography (OCT), characteristic ONH changes with thinning of the neuroretinal rim and glaucomatous cupping as well as corresponding visual field defects without any other ocular or systemic cause for visual field defects [1]. POAG patients were subdivided into a normal and a high pressure group, whereby NPG was defined as POAG with a history of untreated IOP ≤ 21 mmHg. The IOP-lowering medication was not discontinued.

A detailed ophthalmic examination of the anterior eye segment and the fundus as well as 24-hour Goldmann applanation tonometry were performed. The mean of six measurements taken at the following time-points was taken for the analyses: 1, 4, 7, and 10 pm at the slitlamp (Haag-Streit, Koeniz, Switzerland), at midnight in a supine position with handheld Perkins MK3 tonometer (HS Clement Clark Ophthalmic, Haag-Streit UK), and at 7 am again at the slitlamp prior to the application of IOP-lowering medication. Automated perimetry was performed using the Humphrey field analyzer (Swedish interactive threshold algorithm standard 30–2 program; Carl Zeiss Meditec. Dublin, CA, USA). An objective examination of the ONH was performed with confocal laser ophthalmoscopy using the Heidelberg retina tomograph (HRT II, Heidelberg Engineering Inc, Heidelberg, Germany). The HRT parameters rim area and mean retinal nerve fiber layer (RNFL) thickness, as well as the visual field indices mean deviation (MD) and pattern standard deviation (PSD) were analyzed. Furthermore, CCT and anterior segment characteristics were measured with the Pentacam (Pentacam HR 3, Oculus, Wetzlar, Germany) and the inner retinal layers with the OCT glaucoma module (Spectralis^®^, Heidelberg Engineering Inc., Heidelberg, Germany). Subsequently, biomechanical measurements were performed with the ORA and CST.

The ORA uses an infrared beam to monitor the corneal deformation caused by a rapid air pulse. The infrared beam is reflected by the cornea in different deformation states and registered by an optical sensor. From the differences in the acting pressures to achieve defined corneal deformation states, the device calculates the Corneal Hysteresis (CH) and the Corneal Resistance Factor (CRF) [21–23]. In the present study, three consecutive measurements of each eye were obtained and the average (calculated by the ORA software) was used for further analysis.

During CST measurement a two-dimensional cross-section image of the cornea after application of an air pulse is created using a high-speed Scheimpflug camera to measure dynamic response of the cornea and the IOP. This process creates different dynamic corneal response (DCR) parameters describing corneal biomechanical properties [21, 24]. Measurements with the CST were taken once in every eye as previous reports had described reliable and good quality results even after a single measurement [25, 26]. Following DCR parameters were analyzed: the deformation amplitude (DA) ratio max 2 mm, that describes the ratio of the central deformation amplitude and mean peripheral deformation at ± 2mm from apex [27]; the integrated radius, that represent the sum of the concave condition (inverse radius) of the cornea between 1^st^ and 2^nd^ applanation [27]; and the stress-strain index (SSI), that describes elastic properties of the cornea minimizing the influencing factors corneal thickness and intra-ocular pressure [28].

Data were analyzed using the software SPSS (Version 27, IBM Statistics, New York, USA). To correct a possible influence on biomechanical readings caused by IOP, CCT and age [24, 29–31], a group matching for these parameters was performed using the “case control matching” algorithm of the SPSS software. Matching criteria were age (tolerance of 5 years), IOP (tolerance of 2 mmHg), and pachymetry (tolerance of 8 μm). Normal distribution was visually assessed by Q-Q plots. Differences between HPG and NPG patients were compared using the linear mixed model considering the inter-eye correlation [32]. The association to indicators of disease severity (perimetry, HRT) was analyzed using a univariate regression analysis based on the generalized estimating equations and taking into account the correlation between both eyes. Due to multiple testing of five main outcome measures (CH, CRF, DA ratio max 2 mm, integrated radius, and SSI), a P-value of lower than 0.01 was considered as statistically significant.

## Results

Initially, 70 eyes of 42 treated HPG and 139 eyes of 95 treated NPG patients were included. Baseline data of all participants were compared and IOP showed significant group differences (HPG: 15.1 ± 4.7 mmHg, NPG: 13.4 ± 2.3 mmHg, P = 0.004). After group matching, 60 eyes of 38 HPG patients and 103 eyes of 60 NPG patients were included for further analyses. Baseline data of these participants are given in Table 1. According to the glaucoma staging system 2 by Brusini and Filacorda [33], in HPG and NPG a disease severity stage 3 (from 5 stages) was determined.

**Table 1:** Baseline data of high pressure (HPG) and normal pressure glaucoma (NPG) patients after group matching for intraocular pressure, central corneal thickness and age.

	HPG patients		NPG patients		P
	Mean + SD	95% CI	Mean + SD	95% CI	
number eyes/patients	60/38	-	103/60	-	-
age (years)	67.4 ± 8.9	64.4 to 70	67.8 ± 8.3	65.9 to 70.4	0.613
male/female	17/21	-	22/38	-	-
right/left eye	30/30	-	51/52	-	-
BCVA (logMar)	0.09 ± 0.11	-0.08 to 0.04	0.11 ± 0.2	-0.04 to 0.08	0.425
CCT (μm)	542.8 ± 33.8	533.7 to 556.1	530.8 ± 32.9	521.8 to 539.6	0.052
IOP (mmHg)	13.7 ± 2.5	12.9 to 14.5	13.1 ± 2.4	12.5 to 13.7	0.237
number of medications	2.5 ± 1	2.1 to 2.9	2.4 ± 1.3	2.1 to 2.7	0.674
axial length (mm)	23.4 ± 0.9	23.1 to 23.7	23.8 ± 0.8	23.6 to 24.2	0.031
AC depth (mm)	2.54 ± 0.26	2.47 to 2.63	2.66 ± 0.24	2.55 to 2.72	0.14
AC volume (mm^3^)	133.4 ± 29.6	123.9 to 141.7	140.9 ± 25.4	130.2 to 148.3	0.316
MD (dB)	-7.07 ± 6.76	-8.85 to -5.07	-6.43 ± 6.16	-7.97 to -5	0.697
PSD (dB)	6.12 ± 4.55	4.74 to 7.42	7.26 ± 4.53	6.2 to 8.29	0.176
RNFL thickness (mm)	0.19 ± 0.13	0.15 to 0.22	0.17 ± 0.12	0.14 to 0.19	0.387
rim area (mm^2^)	0.99 ± 0.38	0.9 to 1.1	0.94 ± 0.29	0.87 to 1.02	0.376

BCVA: best corrected visual acuity, CCT: central corneal thickness, IOP: intraocular pressure, AC: anterior chamber, MD: mean deviation, PSD: pattern standard deviation. Significance in marked in bold (P < 0.01).

Regarding the IOP-lowering medication, Beta-blockers (HPG: n = 37 (62%); NPG: n = 61 (59%); P = 0.759), prostaglandin derivates (HPG: n = 49 (82%), NPG: n = 88 (85%); P = 0.526), carbonic anhydrase inhibitors (HPG: n = 34 (57%), NPG: n = 64 (62%); P = 0.492), alpha-2-selective adrenergic agonists (HPG: n = 27 (45%), NPG: n = 20 (19%); P = 0.001) and parasympathomimetics (HPG: n = 3 (5%), NPG: n = 14 (14%); P = 0.084) were used.

ORA measurement revealed a significantly higher CRF in HPG than in NPG patients (9.52 ± 2.28 mmHg vs. 8.22 ± 1.68, P < 0.001, Table 2). Also, dynamic corneal response parameters showed significant group differences (Table 2). Inter alia, NPG patients showed a higher integrated radius (8.97 ± 0.96 vs. 8.23 ± 1.3, P < 0.001) in comparison to HPG. The SSI (1.37 ± 0.28 vs. 1.17 ± 0.22, P < 0.001) was increased in HPG compared to NPG.

**Table 2:** Comparison of biomechanical parameters in high pressure (HPG) and normal pressure glaucoma (NPG) patients after group matching.

	HPG patients	NPG patients	P
*Ocular Response Analyzer*			
CH (mmHg)	9.08 ± 1.78	8.58 ± 1.56	0.105
CRF (mmHg)	9.52 ± 2.28	8.22 ± 1.68	**< 0.001**
*Dynamic Scheimpflug-Analyzer Corvis ST*			
DA ratio max 2 mm (mm)	4.5 ± 0.72	4.77 ± 0.53	0.012
integrated radius (mm^-1^)	8.23 ± 1.3	8.97 ± 0.96	**< 0.001**
SSI	1.37 ± 0.28	1.17 ± 0.22	**< 0.001**

CH: corneal hysteresis, CRF: corneal resistance factor, max: maximum, DA: deformation amplitude, SSI: stress-strain index. Significance in marked in bold (P < 0.01).

Regression analysis revealed significant associations between biomechanical parameters and indicators of disease severity. In HPG, SSI correlated significant to RNFL thickness (Table 3). In NPG, the associations were more pronounced and significant dependencies between biomechanical readings and rim area, MD, as well as PSD were detectable. The results are shown in Table 4.

**Table 3:** Regression analysis of the association between corneal biomechanical parameters and functional (visual field) and structural (HRT) indices of glaucoma severity in high pressure glaucoma (HPG) patients using generalized estimating equations.

	RNFL thickness		rim area		MD		PSD	
	Beta	P	Beta	P	Beta	P	Beta	P
CH (mmHg)	-0.012	0.093	0.012	0.591	-0.216	0.673	0.276	0.37
CRF (mmHg)	-0.013	0.088	-0.011	0.748	-0.536	0.244	0.306	0.333
DA ratio max 2 mm (mm)	-0.047	0.067	-0.097	0.321	-1.606	0.225	1.098	0.296
integrated radius (mm^-1^)	-0.021	0.115	-0.04	0.436	-0.455	0.476	0.255	0.656
SSI	**0.159**	**0.008**	-0.096	0.755	1.012	0.765	1.025	0.732

CH: corneal hysteresis, CRF: corneal resistance factor, max: maximum, DA: deformation amplitude, SSI: stress-strain index. Significance in marked in bold (P < 0.01).

**Table 4:** Regression analysis of the association between corneal biomechanical parameters and functional (visual field) and structural (HRT) indices of glaucoma severity in normal pressure glaucoma (NPG) patients using generalized estimating equations.

	RNFL thickness		rim area		MD		PSD	
	Beta	P	Beta	P	Beta	P	Beta	P
CH (mmHg)	-0.008	0.518	**0.077**	**< 0.001**	0.424	0.259	-0.183	0.576
CRF (mmHg)	-0.005	0.501	**0.065**	**< 0.001**	0.771	0.076	-0.427	0.205
DA ratio max 2 mm (mm)	0.002	0.93	-0.091	0.148	**-2.799**	**0.005**	**2.352**	**0.005**
integrated radius (mm^-1^)	-0.012	0.385	-0.051	0.125	**-1.262**	**0.009**	0.978	0.07
SSI	0.08	0.121	0.289	0.054	4.007	0.129	-4.642	0.059

CH: corneal hysteresis, CRF: corneal resistance factor, max: maximum, DA: deformation amplitude, SSI: stress-strain index. Significance in marked in bold (P < 0.01).

## Discussion

Earlier studies reported significant differences in CCT, ORA and CST results between POAG patients and healthy subjects [11, 34–37], but in the current study, we focused on differences between POAG diagnosed as HPG or NPG. Due to the applied matching algorithm, we were able to compare similar patient groups in a sufficient number of cases and with less possible falsifying influences [24, 29–31].

In HPG, the SSI was increased in comparison to NPG, indicating a higher corneal stiffness [28, 38]. Furthermore, in the case of a higher stiffness, a reduction in deformation amplitude ratio max (which was not statistical significant) and integrated radius is expected [39], which was detectable in HPG patients. Although these changes might be caused by a higher IOP [30], the implemented group matching according to IOP, CCT and age, reduces this influence. SSI represents the overall stress-strain behavior of the corneal tissue [28], which was reduced in NPG patients. Similarly, in keratoconus a reduction in corneal stiffness is well known, and a decrease of SSI in moderate keratoconus in comparison to healthy subjects was detected previously [38]. In agreement, Wu et al. [40] and Xu et al. [41] found more deformable corneas in NPG than in HPG and healthy subjects. Furthermore, Vinciguerra and co-workers reported different biomechanics in NPG and HPG. Also shown in this work, corneas of NPG patients had a lower stiffness with a reduction in the stiffness parameter at 1^st^ applanation (SPA1) [36]. The parameter SPA1 differs from the SSI due to it is defined as the resultant pressure during inward movement divided by the corneal displacement from undeformed state to applanation A1. Therefore, SPA1 predominantly represents the elastic component of the corneal resistance to deformation [24]. However, a relationship to corneal thickness and IOP was found in healthy eyes [42]. Simultaneously, ORA measurements revealed a higher CRF in HPG than in NPG, while CH remained not significantly different between the groups. The CH shows corneal viscous damping capacity, whereas the CRF indicates the global resistance against deformation [23, 43]. Therefore, it could be assumed that mainly elastic corneal properties are higher in HPG than in NPG.

These biomechanical differences between HPG and NPG lead to the hypothesis of two disease entities with potentially divergent underlying pathophysiological mechanisms. In agreement, some earlier studies reported differences in the location and the extent of visual field defects [44–47], and the structural damage of the ONH [48] between HPG and NPG patients. On the other hand, some authors showed identical ONH changes [49–51]. Furthermore, genetic differences can be presumed [52, 53], and the peripapillary vessel density differs between HPG and NPG [54]. Lešták et al. reported discrepancies in the results of pattern electroretinogram, pattern visual evoked potentials as well as functional magnetic resonance imaging between HPG and NTG [55]. On this basis, they assumed changes at different points of the visual pathway in both entities [55].

According to the mechanical glaucoma theory, a reduced compliance at the ONH and the LC might be causative. A higher tissue stiffness could increase the susceptibility to IOP fluctuations [14, 18] leading to microcirculatory disturbance, lack of neurotrophic factors due to disorders of axonal transport or direct damage to retinal nerve fibers at the ONH [1, 2]. Conversely, in NPG other mechanisms may be effective, which have also been discussed in the past. Inter alia, these could include retinal glial cell activation, oxidative stress, low cerebrospinal fluid pressure in the subarachnoidal space of the ONH, tissue remodeling with increase in matrix metalloproteinases (MMPs), and loss of neuronal tissue [5, 52, 56].

Earlier studies reported a stiffer TM, LC and peripapillary sclerae in glaucoma [14–17]. The stiffening and increased outflow resistance of the TM may be caused by a growth in extracellular matrix (ECM) with thickening of elastic fibers introduced by a higher resistance against proteolytic degradation and a reduced tissue turnover [14]. Furthermore, according to Albon et al. and Liu et al., age-induced alterations in collagenous and non-collagenous components of ECM of the LC occur, which may further reduce compliance at the ONH [14, 18]. Also, sclera stiffening in POAG was attributed to ECM changes with alterations in collagen fiber alignment and density as well as accumulation of nonenzymatic crosslinking [14]. Another point may be an enhanced accumulation of advanced glycation end products (AGEs) in the ECM of glaucomatous eyes. AGE-induced crosslinking of collagen fibrils was correlated to reduced susceptibility to proteolytic and chemical degradation with a following loss of flexibility [14].

In the context of ECM changes, results by Gramer et al. have to be mentioned [46]. In case of equivalent visual field defects, they found a smaller rim area in NPG than in HPG patients. Assuming that equal visual field defects are associated with the same extent of axonal loss, the authors presumed a stronger decrease of not-neuronal rim tissue in NPG than in HPG. According to Gramer et al., the loss of non-neuronal tissue may be an explanation for the lower IOP tolerance of the ONH in NPG [46]. Thereby, a connective tissue atrophy could be the cause or the consequence of a chronic reduction in ONH perfusion [46, 47]. As previously mentioned, corneal biomechanics may serve as an indicator for structural ONH integrity [19]. However, a conclusion from corneal biomechanical changes to the tissue alterations on a microstructural level seems not to be possible.

A further indication that ECM changes are not limited to TM, LC, ONH and peripapillary sclera [14–17, 46], but further involve the corneal tissue may be the measurement of CCT in POAG. A thinner CCT is a known risk factor for glaucoma progression [3, 57], and it was associated with a smaller rim area and a larger cup volume in POAG [58, 59]. Thinner CCT is found more often in patients with NPG [60]. Furthermore, a more pronounced decrease in CH and CRF in more advanced NPG eyes was reported [34]. On the one hand, CCT reduction is associated with a decreased corneal stiffness [24, 29, 30], but on the other hand, a CCT matching was applied in the current study. Therefore, a more deformable cornea in NPG than in HPG may indicate that the earlier discussed tissue remodeling associated with activation of MMPs may not only take place at the ONH level in NPG [5, 61].

MMPs are a group of proteolytic enzymes degrading ECM components, which are present in all parts of the eye [62]. An imbalance of MMP activity and its inhibitors may be involved in glaucomatous IOP increase [62]. A human cell culture study showed a rise in MT1-MMP expression and a decrease in tissue inhibitor of MMP 2 (TIMP-2) levels caused by IOP increase and stretching of TM cells [63]. In agreement, MMP-9 knockout mice developed ocular hypertension [64]. Other studies demonstrate the role of corneal MMPs, e.g. the involvement of MMP-2 or -9 in corneal ulceration in microbial keratitis [65], corneal scarring [66] and corneal wound healing [67]. Therefore, the higher corneal stiffness reduction in NPG than in HPG might be caused by higher MMP activity leading to enhanced ECM turnover. Moreover, earlier discussed MMP activity at the ONH level [68, 69] could be the cause of a stronger reduction in non-neuronal ONH tissue in NPG as assumed by Gramer et al. [46]. According to Weinreb et al., CCT reduction could be a surrogate marker for MMP activity in glaucoma [62]. The same could be true for corneal biomechanics and differences in the pattern of MMP activity might be a reason for biomechanical differences in HPG and NPG.

This hypothesis is reinforced by the measured associations between indicators of disease severity and biomechanical parameters, which were more pronounced in NPG than in HPG patients. In NPG, ORA as well as CST results correlated to HRT and perimetry indices. For instance, CH and CRF are decreased with smaller rim area. Similarly, an advanced visual field defect (MD reduction, PSD increase) resulted in a higher deformation amplitude ratio in NPG. In both groups, morphological and functional parameters were associated to the parameter SSI. In HPG, RNFL thickness correlated positively to SSI. In NPG, rim area was positively and PSD was negatively related to SSI. These facts lead to the conclusion that a reduced corneal deformation measured by dynamic methods is associated to a more advanced glaucomatous damage in HPG and NPG patients. Therefore, a lower corneal stiffness may be a risk factor or an indicator for a severe disease progression. In accordance, using CST measurements Li et al. found a more deformable cornea in the worse eye of asymmetric NPG patients [12]. In the same manner, Jung et al. reported a quicker progression of perimetric defects in case of a higher deflection amplitude [70] indicating a reduced corneal stiffness [24, 25, 71]. And Park et al. showed lower CH values in more advanced glaucoma classified by HRT measurements [34]. Supporting the hypothesis of concordant changes of the whole globe, Quigley and Cone considered the stiffening of the sclera to be a protective response to IOP effects in glaucoma [72]. In another investigation using confocal scanning laser ophthalmoscopy by Lesk et al., LC movement was detected after IOP reduction. Thereby, individuals with lower corneal thickness showed a higher extent of LC movement and a smaller improvement of neuroretinal rim blood flow after IOP reduction than subjects with higher CCT [73]. Therefore, under the assumption of an equal composition of the ECM of the anterior and posterior ocular tissues, eyes with more deformable corneas may be more susceptible to IOP induced damage of the LC and the peripapillary sclera [12, 36, 57, 74]. This association could explain the connection between a reduced corneal stiffness and advanced glaucomatous damage. However, this is contradicted by other reports, which found higher stiffness of the optic nerve and the peripapillary structures in POAG [75]. Furthermore, Qassim et al. showed that higher SPA1 was associated with a faster rate of RNFL and ganglion cell-inner plexiform layer thinning as well as a greater risk of visual field progression in glaucoma suspects [76].

It may be argued that the credibility of the results is reduced by a potential influence of topic anti-glaucomatous medication. Especially prostaglandins might influence ocular biomechanics [77, 78]. However, no significant difference in treatment regime existed between the study groups. Well known adverse effects of prostaglandins are enophthalmos and deepening of the upper lid sulcus caused by periorbital fat atrophy. The resultant reduction in size and increased density of adipocytes [79] may contribute to reduced orbital compliance in glaucoma. The parameter whole eye movement (WEM) indicates the slow linear motion of the whole eye in the anterior-posterior direction during the applanation process [80]. It can serve as an indicator for retrobulbar tissue changes. However, WEM did not differ between NPG and HPG (data not shown), which contradicts a significant difference in prostaglandin effect between the groups.

Besides topical medication, a further limitation of the current study is the observational, cross-sectional design. Therefore, a longitudinal evaluation of corneal biomechanical risk factors is not possible. Moreover, the necessary group matching reduced the number of participants, and all patients were of Caucasian ethnicity. In consequence, results must be interpreted with caution. The strength of this study lies in the comparison of two very similar cohorts of treated HPG and NPG patients examined under the same conditions.

In conclusion, significant differences in corneal biomechanics were detectable between HPG and NPG patients with generally stiffer corneal properties in HPG. In both groups, a lower corneal stiffness was associated with advanced glaucomatous damage. Therefore, a more deformable cornea may be a risk factor for a severe disease progression in POAG. The measurable differences in corneal biomechanical properties between HPG and NPG lead to the assumption of different underlying pathophysiological mechanisms in both entities.

## Supporting information

S1 Data (XLSX)
